# Erdheim-Chester disease BRAF (-) Diagnosis through cutaneous manifestations and good response with anakinra treatment^☆^

**DOI:** 10.1016/j.abd.2023.02.012

**Published:** 2024-05-31

**Authors:** Juan-Manuel Morón-Ocaña, Amalia Pérez-Gil

**Affiliations:** Dermatology, Hospital Universitario Virgen de Valme, Sevilla, Spain

Dear Editor,

Erdheim-Chester disease (ECD) is a very rare non-Largerhans systemic histiocytosis of unknown origin. Approximately 550 cases have been described in literature since its first publication.^1^

It is characterized by xanthogranulomatous infiltration of different tissues with numerous foam histiocytes. The disease can be very heterogeneous, ranging from indolent cases to a life-threatening multisystemic disease with possible bone, neurological, skin, hypothalamic-pituitary, pulmonary and renal infiltration.

Long bones are affected in more than 90% of the cases. They have a typical infiltration pattern which consists of symmetric osteosclerosis patches in the diaphyses without epiphyses affection.^1^

The most frequent cutaneous manifestations are xanthelasma-type lesions that occur in 25 %–30 % of patients and which can become very deforming. Sometimes skin manifestations precede the systemic clinic.^2^

Historically, ECD lacked effective treatments. The recent discovery that more than 60% of patients with ECD have the BRAF^V600E^ mutation has led to the indication of targeted therapies (MEK and BRAF inhibitors).^3^, ^4^ However, when BRAF mutation is negative, there have not been prospective controlled therapeutic trials to compare treatments.

We have followed up on a 55-year-old man for a decade. In 2010, at 45 years old, the patient made a consultation about cutaneous facial thickening and progressive facial deformity with difficulty in oral opening. He presented exophthalmos with yellow-orange skin, large telangiectasias, and lower eyelid big bags (Fig. 1).

**Fig. 1:**
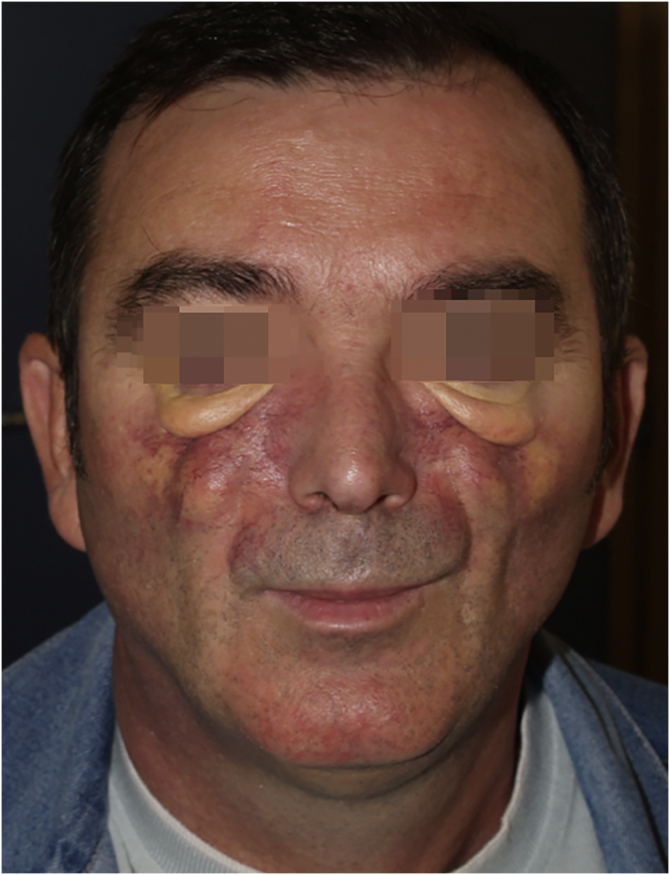
Physical appearance 2010: cutaneous facial thickening and progressive facial deformity, exophthalmos with yellow-orange skin, large telangiectasias and lower eyelid big bags.

The cutaneous manifestations were very disfiguring. After multiple facial biopsies, the diagnosis of ECD was reached after visualizing an extensive infiltration by foam macrophages (xanthic cells, CD68 +, CD163 +, S100-, CD1a−) (Fig. 2).

**Fig. 2:**
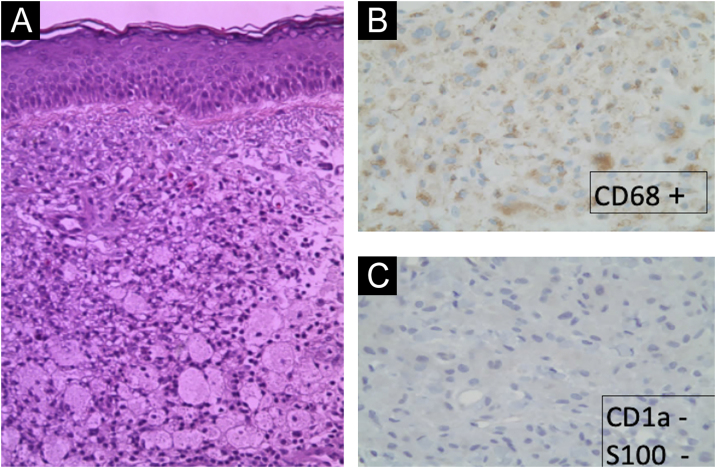
(A) Hematoxylin & eosin (×20): extensive infiltration by foam macrophages. (B) IHQ (×10): xantic cells CD68+. (C) IHQ (×10); xantic cells S100−, CD1a−.

Initially, the patient was treated with high doses of corticosteroids (prednisone 1 mg/kg/day) and interferon. Since 2016, he has presented an increase in his basal dyspnea and a progressive elevation of acute phase reactants. It was decided to extend the study with complementary tests. Among them, splenomegaly and pulmonary infiltrate with an interstitial pattern were observed without significant bone findings. The patient presented a negative molecular study for mutations in the regions of the NRAS and BRAF genes.

In 2019, it was decided to prescribe anakinra 100 mg subcutaneously daily as an off-label indication^5^ due to the lack of response to the combined therapy. In case of no clinical response, anakinra could be increased to 200 mg/day.^6^

Since then, the patient has improved progressively without requiring an increase in anakinra dose. Nowadays, the patient is asymptomatic. The skin filling has been remitted but it has required a blepharoplasty to correct the redundant skin and multiple sessions of dye-pulsed light for the treatment of facial vascular lesions (Fig. 3).

**Fig. 3:**
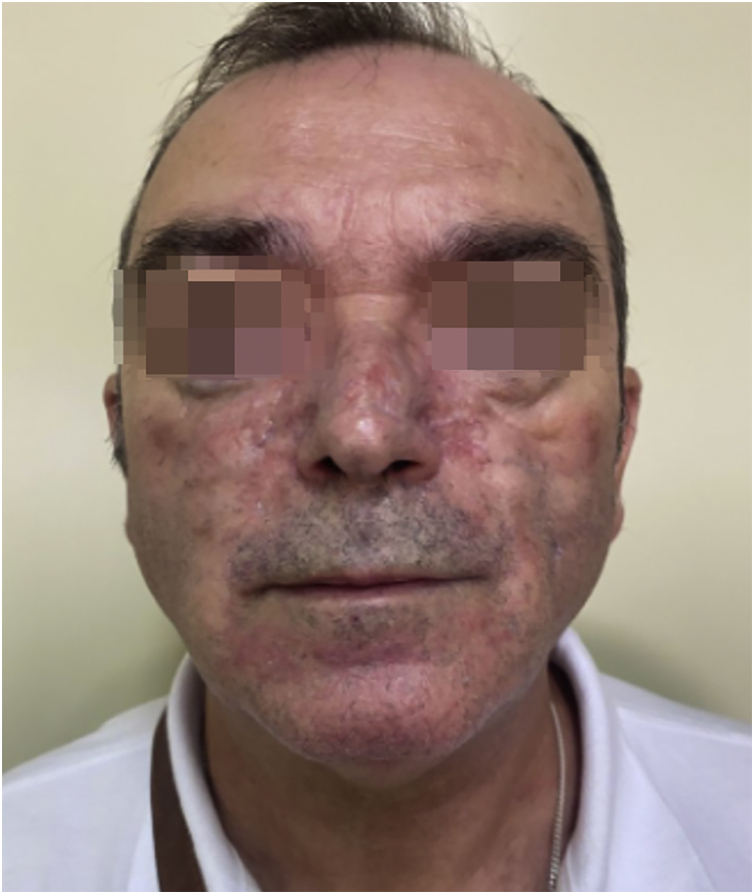
Physical appearance 2020: The skin infiltration has remitted after anakinra, blepharoplasty and dye-pulsed light.

We have presented a rare multisystemic disease of unknown etiology, whose diagnosis and treatment continue to be a challenge today.

The bone clinic, the main manifestation of the disease, is always absent. In this case, the cutaneous manifestations stand out from the beginning, even before the systemic symptoms.

Although the discovery of the involvement of the BRAF pathway in ECD has led to a revolution in its treatment, in our case it is also absent. Due to the ultra-rareness of ECD, there have not been any prospective controlled clinical trials to compare treatments.

Proinflammatory cytokines, such as IL-1, IL-6, and TNF-alpha, are strongly increased in ECD lesions. These findings suggested that inhibition of the IL-1 pathway could be a promising therapeutic area for ECD treatment.^5^

Our experience supports the use of anakinra, an IL-1 receptor antagonist, as a therapeutic option for ECD when the BRAF mutational study is negative.

Despite the progress in understanding the underlying pathogenesis and biology of ECD, we believe that more efforts are needed in the study of the disease.

## Financial support

None declared.

## Authors’ contributions

Juan-Manuel Morón-Ocaña: Preparation and writing of the manuscript and critical literature review.

Amalia Pérez-Gill: Approval of the final version of the manuscript and manuscript critical review.

## Conflicts of interest

None declared.
